# African swine fever virus AP endonuclease is a redox-sensitive enzyme that repairs alkylating and oxidative damage to DNA

**DOI:** 10.1016/j.virol.2009.04.021

**Published:** 2009-07-20

**Authors:** Modesto Redrejo-Rodríguez, Alexander A. Ishchenko, Murat K. Saparbaev, María L. Salas, José Salas

**Affiliations:** a Centro de Biología Molecular “Severo Ochoa” (Consejo Superior de Investigaciones Científicas/Universidad Autónoma de Madrid), Universidad Autónoma de Madrid, C/Nicolás Cabrera 1, 28049 Madrid, Spain; b Groupe «Réparation de l'ADN», CNRS UMR 8126, Univ. Paris-Sud, Institut de Cancérologie Gustave Roussy, F-94805 Villejuif Cedex, France

**Keywords:** African swine fever virus, BER, NIR, Redox regulation, Genotoxic agents, DNA damage

## Abstract

African swine fever virus (ASFV) encodes an AP endonuclease (pE296R) which is essential for virus growth in swine macrophages. We show here that the DNA repair functions of pE296R (AP endonucleolytic, 3′ → 5′ exonuclease, 3′-diesterase and nucleotide incision repair (NIR) activities) and DNA binding are inhibited by reducing agents. Protein pE296R contains one intramolecular disulfide bond, whose disruption by reducing agents might perturb the interaction of the viral AP endonuclease with the DNA substrate. The characterization of the 3′ → 5′ exonuclease and 3′-repair diesterase activities of pE296R indicates that it has strong preference for mispaired and oxidative base lesions at the 3′-termini of single-strand breaks. Finally, the viral protein protects against DNA damaging agents in both prokaryotic and eukaryotic cells, emphasizing its importance *in vivo*. The biochemical and genetic properties of ASFV AP endonuclease are consistent with the repair of DNA damage generated by the genotoxic intracellular environment of the host macrophage.

## Introduction

The maintenance of DNA integrity is essential not only for the survival of cellular organisms but also viruses. Various exogenous and endogenous agents can induce DNA damage which leads to mutations, DNA replication blocks and genetic instability (Alberts, 2002; Lindahl, 1993). A large variety of modifications in DNA are caused by these agents, including abasic sites, single and double strand DNA breaks, and alkylated and oxidized bases. Eukaryotic, prokaryotic and archaea organisms possess a range of highly conserved DNA repair pathways. The mammalian Base Excision Repair (BER) is initiated by a DNA glycosylase that excises damaged or inappropriate bases, such as uracil, generating an abasic site that is subsequently incised by an apurinic/apyrimidinic (AP) endonuclease, giving rise to a 3′-OH group and a 5′-deoxyribose phosphate (dRP) (David et al., 2007; Friedberg and Wood, 1996; Lindahl, 1990). This dRP group is eliminated by the dRP lyase activity of the specific BER DNA polymerase during the gap-filling step. Finally, a DNA ligase seals the nick. In the alternative to BER, Nucleotide Incision Repair (NIR) pathway, the same AP endonucleases incise DNA strands containing various oxidized bases, as well as α-anomeric dA, dT and dC nucleotides, by cleaving the DNA backbone on the 5′ side of the lesion and generating a nick with 3′-OH termini (Ishchenko et al., 2006). In addition, oxidizing agents induce DNA strand breaks with blocking groups such as 3′-phosphate (3′-P) and/or 3′-phosphoglycolate (3′-PG) that should be removed by the 3′-phosphatase and 3′-phosphodiesterase activities of AP endonucleases. Furthermore, AP endonucleases display 3′ → 5′ exonuclease activity (Chou and Cheng, 2002; Kerins et al., 2003) that improves the fidelity of DNA repair and may also be involved in alternative repair pathways for misincorporated oxidized dNTPs (Ishchenko et al., 2005).

AP endonucleases are classified into two families according to their homology to *E. coli* AP endonucleases: exonuclease III or Xth and endonuclease IV or Nfo (Demple, 1996). African swine fever virus (ASFV) AP endonuclease is an Nfo-like protein encoded by open reading frame E296R (Lamarche and Tsai, 2006; Redrejo-Rodríguez et al., 2006; Yáñez et al., 1995). Intriguingly, ASFV does not encode any DNA glycosylase, raising the possibility that the viral AP endonuclease and the repair DNA polymerase (pol X) might eliminate DNA damage in an alternative viral BER pathway (Garcia-Escudero et al., 2003). Protein pE296R contains AP endonuclease and 3′ → 5′ exonuclease activities (Lamarche and Tsai, 2006; Redrejo-Rodríguez et al., 2006). Recently, 3′-phosphodiesterase, 3′-phosphatase and weak NIR activities have also been characterized in pE296R (Lamarche and Tsai, 2006). Sequence alignment and 3D structure prediction by homology modelling suggest that protein pE296R, like Nfo protein, is a metalloenzyme with a trinuclear Zn^2+^ core. Moreover, the *E296R* gene is required for viral growth in swine macrophages, the natural host cell of ASFV, presumably due to its DNA repair functions (Redrejo-Rodríguez et al., 2006).

Cells of the immune system, including monocytes, macrophages and neutrophils, have been reported to produce H_2_O_2_, O_2_^•−^ and NO• as a response to certain viruses (Klebanoff and Coombs, 1992; Suzuki et al., 1997). These immune cells are susceptible to infection by ASFV (Fernandez et al., 1992) and, therefore, the virus may undergo an oxidative stress during its replication in the cytoplasm (García-Beato et al., 1992) which would generate lesions in the viral DNA consisting of oxidized bases and single-strand breaks bearing 3′-blocking termini. In both prokaryote and eukaryote kingdoms, cellular resistance to H_2_O_2_ exposure has been attributed to the 3′-repair diesterase and 3′ → 5′ exonuclease activities of AP endonucleases (Demple et al., 1986; Unk et al., 2001; Wilson et al., 1995). Similarly, the H_2_O_2_-induced oxidative injuries in the replicating and/or already replicated ASFV genomes might be removed by the 3′-repair activities of the viral AP endonuclease.

Interestingly, the major human AP endonuclease (APE1) is sensitive to the redox environment, since its AP endonuclease activity is inhibited under highly oxidizing conditions (Kelley and Parsons, 2001), while in the case of the *E. coli* Nfo protein, it has been shown that the 3′ → 5′ exonuclease activity is inhibited by the reducing agent DTT (Kerins et al., 2003).

These observations led us to examine whether the different activities of ASFV AP endonuclease are sensitive to redox agents. In addition, we have further characterized the NIR and 3′ → 5′ exonuclease activities with regard to base pair preferences of protein pE296R. Furthermore, to elucidate the *in vivo* role of the viral AP endonuclease, we have first examined complementation of the AP endonuclease-deficient *E. coli* strain to chronic exposure of H_2_O_2_, methylmethanesulfonate (MMS) and *tert*-butyl hydroperoxide (*t*-BuO_2_H) by expression of the pE296R and Nfo proteins. Second, we tested the effect of these genotoxic agents on cells infected with wild type ASFV and a mutant virus lacking the *E296R* gene (Redrejo-Rodríguez et al., 2006). Our results strongly suggest that the ASFV AP endonuclease is an enzyme adapted to repair diverse DNA damages in an oxidizing intracellular environment.

## Results and discussion

### The AP endonucleolytic activity of pE296R is inhibited by reducing agents

**Fig. 1:**
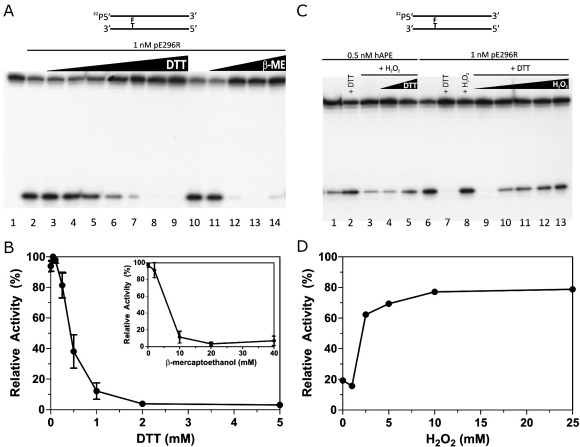
Redox-dependent modulation of ASFV AP endonucleolytic activity. AP endonuclease assays were carried out with 5′-[^32^P]-labelled RT-THF:CompT oligonucleotide duplex in the presence of 1 nM ASFV pE296R or 0.5 nM APE1, as indicated. The configuration of the substrate is indicated above (F stands for THF). (A) Effect of reducing agents. PAGE analysis of the reaction products. Lane 1, no enzyme; lanes 2–9, 0, 0.05, 0.1, 0.25, 0.5, 1, 2 and 5 mM DTT, respectively; lanes 10–14, 0, 2, 10, 20 and 40 mM β-ME, respectively. (B) Graphic of inhibition of ASFV pE296R-catalyzed AP endonuclease activity by DTT and β-ME (inset). The 100% activity corresponds to the maximal enzyme activity obtained with 0.05 mM DTT or in absence of β-ME in the inset. Each point is the mean of two independent experiments. The error bar indicates standard deviation. (C) Reversion of DTT inhibition by H_2_O_2_. PAGE analysis of the reaction products. The proteins were first incubated with or without DTT (20 min, 4 °C) and then with or without H_2_O_2_ (10 min, 4 °C) before performing the incision experiment. Lanes 1 and 6, without DTT or H_2_O_2_; Lanes 2 and 7, 5 mM DTT; lanes 3 and 8, 10 mM H_2_O_2;_; lanes 4 and 5, 10 mM H_2_O_2_ and 1 and 10 mM DTT, respectively; lanes 9–13, 5 mM DTT and 1, 2, 5, 10 and 25 mM H_2_O_2_, respectively. (D) Graphic of reversion by H_2_O_2_ of the DTT inhibition of ASFV pE296R AP endonuclease activity. The 100% activity corresponds to the maximal enzyme activity obtained in the presence of 0.05 mM DTT.

In previous studies, dithiothreitol (DTT) has been frequently used in reaction buffers for various AP endonucleases of different origins (Levin et al., 1988; Salas-Pacheco et al., 2003; Wilson, 2005), including ASFV AP endonuclease (Lamarche and Tsai, 2006; Redrejo-Rodríguez et al., 2006). However, it has been shown that DTT strongly inhibits the 3′ → 5′ exonuclease activity of Nfo (Kerins et al., 2003). Since it was possible that reducing agents might also inhibit any of the different repair activities of pE296R protein, we performed, as a first approach, AP endonuclease assays with the viral enzyme. The assays were carried out with an AP site-containing duplex oligonucleotide, in the presence of different concentrations of DTT and β-mercaptoethanol (β-ME) (Fig. 1). The AP endonucleolytic activity of pE296R shows a slight increase in the presence of a low DTT concentration (50 μM, Fig. 1A, lane 3 and Fig. 1B), but at 1 mM and higher DTT concentrations AP site incision was strongly inhibited with less than 10% of the activity remaining (Fig. 1A, lanes 7–9, and Fig. 1B). With β-ME, almost complete inhibition is observed at concentrations of 10 mM and higher (Fig. 1A, lanes 12–14 and Fig. 1B, inset).

We calculated afterwards the kinetic parameters for AP endonuclease activity of pE296R in the presence and absence of 0.5 mM DTT (see details in Materials and methods). In the presence of DTT the *K*_M_ value increased moderately, from 120 ± 20 nM to 170 ± 25 nM, while the catalytic efficiency (*k*_cat_/*K*_M_) was reduced almost 3-fold: 48.7 min^− 1^ μM^− 1^ without DTT versus 17.3 min^− 1^ μM^− 1^ with DTT. This difference confirms the negative effect of DTT on the catalytic activity of the viral AP endonuclease.

To see whether the effect of reducing agents on AP endonuclease activity of protein pE296R is permanent or it is reversible under oxidizing conditions, we studied the effect of H_2_O_2_ on the DTT-reduced pE296R protein. Human APE1 is inhibited by H_2_O_2_ and diamide in a reversible way by DTT (Masuda et al., 1998); therefore, we included the effect of H_2_O_2_ and DTT on the purified APE1 as a control. In this experiment, we pre-incubated the proteins with or without DTT (20 min at 4 °C) and then with or without H_2_O_2_ (10 min at 4 °C) before performing the incision assays (Figs. 1C and D). The incision activity of APE1 was stimulated by DTT and inhibited by H_2_O_2_, this inhibition being partially reverted by DTT (Fig. 1C, lanes 1–5). In the case of ASFV AP endonuclease, we found that the AP endonuclease activity was not affected by H_2_O_2_ (Fig. 1C, lane 8) but, as expected, was strongly inhibited by DTT (Fig. 1C, lane 7). This inhibition by DTT was reverted by H_2_O_2_ in a dose-dependent manner (Fig. 1C, lanes 9–13 and panel D). However, no reversion was found by incubation with Zn^2+^ cation (not shown), ruling out the possibility that the DTT-dependent inhibition found might be due to the formation of a Zn^2+^–DTT complex, as has been shown for other zinc-containing enzymes (Cornell and Crivaro, 1972). Also, the inhibition by DTT was not reverted by other divalent cations, including Co^2+^, Ca^2+^ and Ni^2+^.

### The 3′ → 5′ exonuclease, NIR and 3′-repair diesterase activities of pE296R are sensitive to DTT

**Fig. 2:**
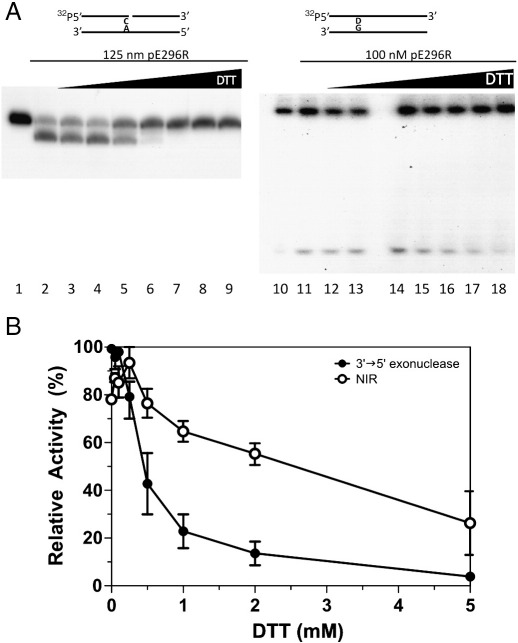
Inhibition of ASFV pE296R 3′ → 5′ exonuclease and NIR activities by DTT. Assays for 3′ → 5′ exonuclease were carried out with 5′-[^32^P]-labelled ExoC/RexA oligonucleotide duplex, while NIR activity was analyzed with 5′-[^32^P]-labelled RT-DHU/CompG. The configuration of the substrate is shown above (D stands for DHU) Enzyme concentration is shown for each group of samples. (A) PAGE analysis of 3′ → 5′ exonuclease degradation and NIR cleavage products. Lanes 1 and 10, no enzyme; lanes 2–9 and 11–18, 0, 0.05, 0.1, 0.25, 0.5, 1, 2 and 5 mM DTT, respectively. Note that an empty lane separates lanes 13 and 14. (B) Graphic representation of inhibition of exonuclease and NIR activities by DTT. The 100% activity corresponds to the maximal activity obtained in each experiment. Each point is the mean of two independent experiments. The error bar indicates standard deviation.

To further examine the redox modulation of protein pE296R *in vitro*, we also measured the effect of DTT on the 3′ → 5′ exonuclease, NIR, 3′-phosphatase and 3′-phosphodiesterase activities of the ASFV protein. DTT inhibited the exonuclease activity of pE296R (Fig. 2). Moreover, incubation with H_2_O_2_ turned back the DTT effect (data not shown).

Inhibition of NIR activity by reducing agents was examined on 5,6-dihydrouracil (DHU)-containing duplex oligonucleotide. In the absence of DTT, pE296R shows significant NIR activity (compare lanes 10 and 11 of Fig. 2A), in agreement with a previous report (Lamarche and Tsai, 2006). At low DTT concentrations, NIR activity is slightly affected (Fig. 2A, lanes 12–14), but strongly inhibited at higher DTT concentrations in a dose-dependent manner (Fig. 2A, lanes 15–18, and 2B). We have used both 5′ and 3′-labelled substrates to verify that the incision was indeed on the 5′-side of the damaged nucleotide (data not shown). It should be stressed that the pE296R protein was purified from *E. coli* BH110 strain, ruling out the possibility of contamination by a bacterial AP endonuclease. We also found a strong inhibition of 3′-phosphatase and 3′-phosphodiesterase activities of pE296R (not shown).

### The DNA binding capacity of pE296R is affected by reducing agents

**Fig. 3:**
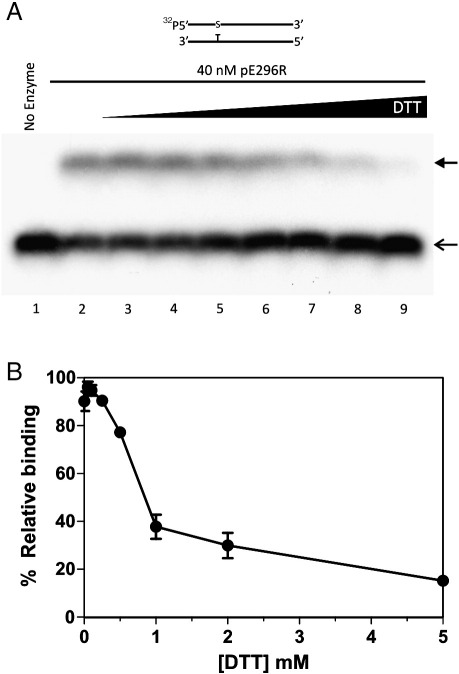
Inhibition of pE296R DNA binding by DTT. Electrophoretic mobility shift assay (EMSA) was carried out with 5′-[^32^P]-labelled RT-S/CompT oligonucleotide duplex. The configuration of the substrate is indicated above (S stands for a 5′-phosphothioate-bound AP site). (A) Non-denaturing gel electrophoresis. Lane 1, no enzyme; lanes 2–9, in the presence of 0, 0.05, 0.1, 0.25, 0.5, 1, 2 and 5 mM DTT, respectively. The bands corresponding to free DNA are indicated with an open arrow and bound DNA with a black arrow. (B) Graphic representation of the DNA binding inhibition by DTT. The 100% binding corresponds to the maximal band shift obtained in the presence of 0.05 mM DTT. Each point is the mean of three independent experiments. The error bar indicates standard deviation.

To examine the mechanism of inhibition of DNA repair activities of the viral protein by reducing agents, we measured pE296R binding to an AP site-containing DNA duplex using electrophoretic mobility shift assay (EMSA) (Fig. 3). We used an oligonucleotide with a 5′-phosphothioate bound to the abasic sugar, to block AP endonuclease incision activity. About 45% of the substrate (open head arrow) in the absence of DTT was in complex with the enzyme and produced a shifted band (black head arrow) (Fig. 3A, lane 2 and B). The increase of DTT concentration produced a decrease of protein–DNA complex formation (Fig. 3A, lanes 3–9 and B). Moreover, the patterns of inhibition of endonuclease activity and DNA binding are similar, suggesting that the inhibition of catalytic activity of the viral protein by reducing agents is due to impairment of the recognition/binding step. This is further supported by the difference in *K*_M_ values at 0 and 0.5 mM DTT.

### Purified native pE296R contains disulfide bonds

**Fig. 4:**
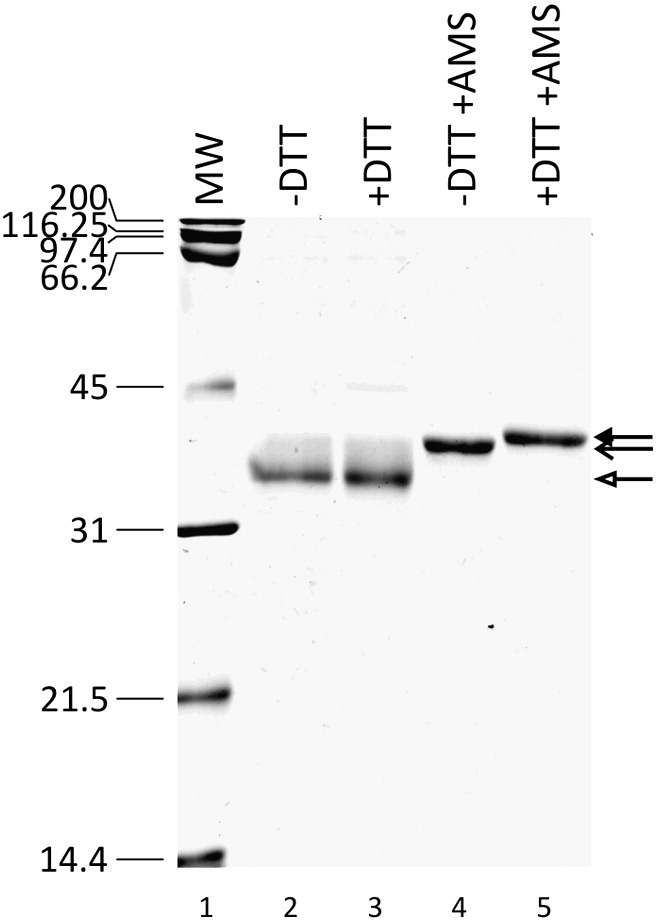
Disulfide bond analysis of the native and reduced pE296R protein. Migration pattern of the pE296R protein samples on 15% SDS-PAGE. Reduction and alkylation were performed with 100 mM DTT and 15 mM AMS, respectively. Lane 1, molecular size markers in kDa; lane 2, native form of pE296R; lane 3, reduced form of pE296R; lane 4, alkylated form of native pE296R; lane 5, alkylated form of reduced pE296R. The white arrow indicates the position of non-alkylated pE296R, the open arrow the position of alkylated form of native pE296R, the black arrow the position of alkylated form of reduced pE296R.

The ASFV protein pE296R sequence contains 7 cysteine residues. This led us to consider whether the inhibitory effect of DTT might be related to the rupture of one or more potential disulfide bonds. To examine whether pE296R contains disulfide bonds, we incubated the viral protein with or without DTT and then treated with the alkylating agent 4-acetamido-4′-maleimidylstilbene-2,2′-disulfonic acid (AMS, 0.5 kDa). As shown in Fig. 4, after alkylation, both reduced or non-reduced pE296R migrated in SDS-PAGE more slowly than non-alkylated pE296R (lanes 4–5 versus 2–3) suggesting the presence of free cysteine residues in the non-reduced form of the protein. Alkylated proteins migrated as single species indicating that the protein sample was in the same oxidation state. Importantly, after alkylation the non-reduced form migrated faster than the reduced form of pE296R. Based on interpolation of the migration distance of the molecular weight markers (lane 1), we calculated an empirical size for alkylated proteins of 38.7 and 39.8 kDa for the non-reduced and reduced preparations, respectively (with a *R*^2^ of 0.85 and an estimated error of ± 0.15 kDa). The difference of size in the two alkylated bands was 1.1 ± 0.15 kDa suggesting that the reduced pE296R protein contains 2 more AMS molecules than the non-reduced pE296R protein. Based on this result, we propose that the native pE296R protein has one disulfide bond and that break-up of this cysteine-cysteine bond by reducing agents might lead to the loss of DNA binding and enzymatic activities of pE296R.

Although the *in vivo* significance of these observations is not known at present, it is tempting to speculate that the presence of a disulfide bond in the viral AP endonuclease may provide a mechanism for regulating the enzyme activity in the infected cells by inducing or breaking this bond. In relation to this possibility, it is interesting to mention that ASFV codes for a sulfhydryl oxidase (Rodríguez et al., 2006), which might be involved in the formation of the disulfide bond. Further work will be required to verify this possibility and determine the state of the pE296R disulfide bond during the viral infection, as well as to ascertain its contribution to the viral AP endonuclease functions *in vivo*.

### The NIR and 3′-end clearing activities of pE296R suggest its role in repairing oxidative damage to DNA

**Fig. 5:**
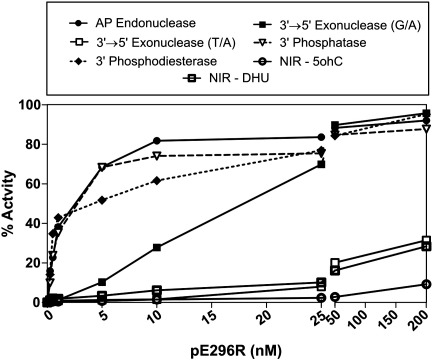
DNA repair activities of pE296R as a function of protein concentration. AP endonuclease activity was measured on THF⋅G (closed circle), NIR activity on 5ohC⋅G (open bold circle) and DHU⋅G (open bold square) oligonucleotide duplexes and 3′ → 5′ exonuclease on mismatched G⋅A (closed square) and matched T⋅A (open square), 3′-phosphatase on p⋅A (open triangle), 3′-phosphodiesterase on THF⋅A (closed diamond) nicked oligonucleotide duplexes. Each point represents the percentage of reaction product measured by gel densitometry.

Our finding that reducing agents inhibit pE296R *in vitro* led us to re-examine some of the biochemical properties of the recombinant protein in the absence of reducing agents. First, we measured enzyme saturation curves for all activities of the ASFV AP endonuclease (Fig. 5). The saturation profile for the different activities can be classified in two groups. AP endonuclease, 3′-phosphatase and 3′-phosphodiesterase reach the maximum activity at 10 nM enzyme concentration. The AP endonuclease activity was the most efficient, followed closely by 3′-phosphatase and 3′-phosphodiesterase activities of the protein (Fig. 5). In contrast, the 3′ → 5′ exonuclease on a correctly paired substrate and NIR activity on DHU and 5-hydroxycytosine (5ohC) were very low and required much higher enzyme concentrations to cleave 5–20% of DNA substrate. The exonuclease activity on a 3′-mismatched substrate showed an intermediate profile, being, as described previously (Redrejo-Rodríguez et al., 2006), much more efficient than on the substrate with regular base pair, which further supports the proofreading role of its exonuclease activity. To further characterize the substrate specificity of pE296R we measured its nucleotide incision activity on full duplex oligonucleotides containing different damaged nucleotides, such as α-deoxyadenine (α-dA), 3,*N*^4^-ethenocytosine (ɛC) and 1,*N*^6^-ethenoguanine (ɛA). αdA constitutes a major adduct detected in irradiated DNA under anoxic condition, whereas etheno(ɛ)-adducts ɛC and ɛA are generated in DNA by products of lipid peroxidation. In contrast with the results obtained with *E. coli* Nfo, assayed in parallel as a positive control, no activity was found with pE296R on all the substrates tested (data not shown) suggesting that the ASFV protein is not a classic NIR endonuclease and has limited substrate specificity as compared to *E. coli* Nfo, yeast Apn1 and human APE1. However, the activity of pE296R on DHU and 5ohC implies a partial involvement of the viral enzyme in the NIR pathway, a conclusion that is supported by the complementation studies reported below.

**Fig. 6:**
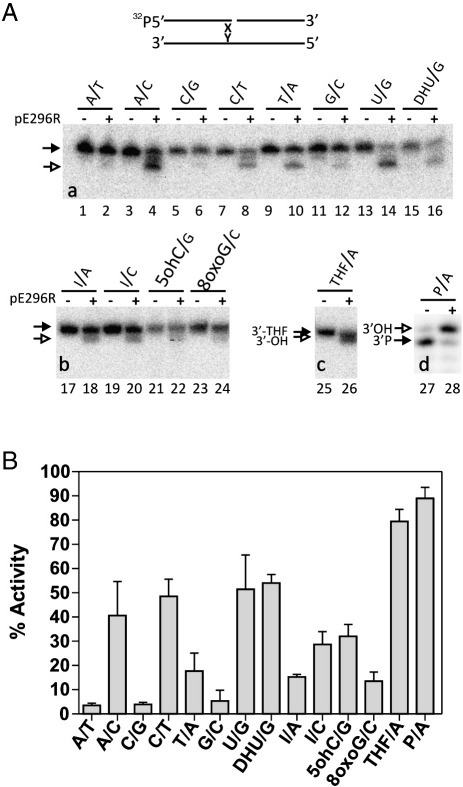
Substrate specificity of pE296R-catalyzed 3′-repair activities. (A) The 5′-[^32^P]-labelled X⋅Y nicked oligonucleotide duplex, schematically represented on the top, was incubated in the presence (+) or absence (–) of 25 nM pE296R. “a” and “b” show the products of 3′ → 5′ exonuclease degradation, “c” 3′−phosphodiesterase and “d” 3′-phosphatase activity. The black and white arrows indicate substrate and product of reaction, respectively. (B) Graphic representation of pE296R activity on various DNA substrates. The 100% activity represents complete conversion of the substrate to product. Each point is the mean of two independent experiments. The error bar indicates standard deviation.

It was previously demonstrated that the AP endonucleases can cleanse DNA 3′-termini from oxidized bases misincorporated during DNA replication or generated directly in the DNA by ROS (Ishchenko et al., 2005; Parsons et al., 2005). To examine whether the viral 3′ → 5′ exonuclease activity may also act directly on damaged 3′-ends, we assayed this activity on nicked duplex DNA containing regular and a broad range of damaged and mismatched base pairs at the 3′-side of the single-strand break. As shown in Fig. 6, DNA substrates containing 3′-terminal mismatches A/C and C/T and damaged bases were very efficiently cleaned up by the pE296R 3′ → 5′ exonuclease activity. In contrast, the matched pairs A/T, G/C, and C/G were very weakly degraded. Nevertheless, the matched base pair T/A was more efficiently processed by pE296R as compared to A/T (20% versus 5%). In keeping with this, the 3′-terminal damaged pyrimidines (U, DHU and 5ohC) were removed with higher efficiency than damaged purines inosine (I) and 7,8-dihydro-8-oxoguanine (8oxoG) (Fig. 6), possibly because of the size of the enzyme's active site pocket which may accommodate more easily smaller nucleotides. The efficient removal of 3′-terminal oxidized nucleotides suggests that ASFV pE296R 3′ → 5′ exonuclease activity may act not only as a backup for DNA polymerase proofreading system, but also as an alternative damage-cleansing function for misincorporated damaged nucleotides, as it was proposed for host AP endonucleases (Ishchenko et al., 2005; Parsons et al., 2005).

As expected, the highest activity of pE296R was observed on DNA substrates containing 3′-blocking groups (tetrahydrofuran (THF) and phosphate). The DNA strand breaks generated by ROS are refractory to DNA repair synthesis because of sugar and/or phosphate residues that block their 3′ termini. Removal of the 3′-blocking groups restores normal nucleotide primers with 3′-OH termini which is an essential step in the repair of these ROS-induced DNA strand breaks. The highly efficient 3′-repair diesterase activities of pE296R indicate their role in the repair of single-strand breaks in viral DNA to remove phosphates and phosphoglycolates at 3′-termini.

### Genotoxic challenges reveal the importance of pE296R DNA repair functions *in vivo*

**Fig. 7:**
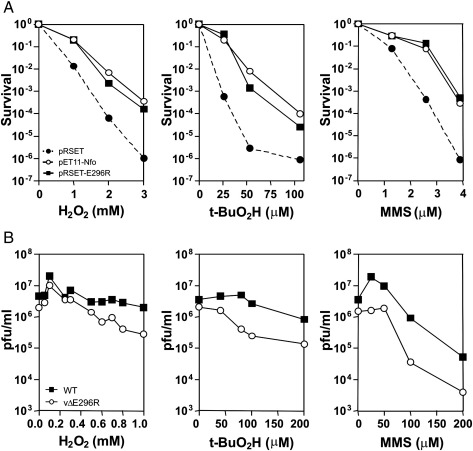
*E296R* gene expression-induced protection against drugs. (A) Drug sensitivity of *E. coli* strains carrying the *E296R* and *Nfo* genes. Graphs correspond to the sensitivity of *E. coli* BH110 (*xth nfo*) carrying pRSET_A_ (black circle), pET11_A_-Nfo (white circle) and pRSET_A_-E296R (black square) to H_2_O_2_, *t*-BuO_2_H and MMS exposure. (B) Titers of BA71V wild type (black square) and vΔE296R (white circle) virus infections in the presence of H_2_O_2_, *t*-BuO_2_H and MMS.

To address the biological role of the various activities of ASFV AP endonuclease, we used two different approaches. First, we carried out complementation assays for the *E. coli xth nfo* strain (BH110) exposed to various genotoxic agents. Among the oxidizing agents, H_2_O_2_ is produced by the host immune cells infected with certain viruses and therefore might generate oxidative lesions in the ASFV DNA (Israel and Gougerot-Pocidalo, 1997; Suzuki et al., 1997). It would thus be of particular interest to determine the capacity of the pE296R protein to confer protection to bacteria cells against H_2_O_2_ treatment. We used Nfo as a positive control for drug-sensitivity complementation of BH110 (Cunningham et al., 1986). As shown in Fig. 7A, the plasmids encoding pE296R and Nfo proteins, in contrast to empty vector, conferred resistance to BH110 cells when exposed to H_2_O_2_. Protection by the ASFV *E296R* gene against *t*-BuO_2_H and MMS was also observed (Fig. 7A), confirming previous results in *E. coli* (Lamarche and Tsai, 2006). Altogether, these data suggest that the viral AP endonuclease can repair 3′-blocking groups, oxidized bases and AP sites *in vivo*. Interestingly, the protection against H_2_O_2_ and MMS provided by both pE296R and Nfo endonucleases was very similar, suggesting highly efficient properties of the viral AP endonuclease to counteract DNA damage *in vivo*. It has been shown that expression of Nfo-G149D point mutant, that lacks the NIR activity but maintains the AP endonuclease activity *in vitro*, can complement double *nfo xth* mutant to the alkylating agent MMS challenge but not to *t*-BuO_2_H, suggesting that the NIR activity is involved in the removal of *t*-BuO_2_H-induced DNA damage (Ishchenko et al., 2006) Thereby, although *in vitro* the ASFV AP endonuclease-catalyzed NIR activity is weaker compared to other DNA repair functions, the protection of bacterial cells by pE296R against *t*-BuO_2_H exposure (Fig. 7A, central panel) suggest a potential role of pE296R in the repair of oxidative DNA base lesions *via* the DNA glycosylase-independent NIR pathway *in vivo*. These results suggest that the viral AP endonuclease possesses all the DNA repair functions of the host homologous AP endonuclease to remove damage to cellular DNA induced by various genotoxic agents.

To validate the results found in bacteria in the context of viral infections, we infected cells in the presence of the same genotoxic agents. We had previously shown that the replication of an ASFV mutant lacking the *E296R* gene (vΔE296R) was severely impaired in swine macrophages, but not in Vero cells (Redrejo-Rodríguez et al., 2006). We therefore tested the effect of exposure with H_2_O_2_, *t*-BuO_2_H and MMS on the replication of wild type ASFV and vΔE296R mutant virus in Vero cells. As shown in Fig. 7B, exposure to small concentrations of H_2_O_2_ and MMS increased the viral titers, but higher concentrations of all three agents decreased viral production in a dose-dependent manner. Importantly, in control experiments, exposure to the drugs, at the concentrations used, did not affect survival of the uninfected cell (not shown). Wild type virus replication showed very low sensitivity to H_2_O_2_ and *t*-BuO_2_H, but was impaired in the presence of MMS. In contrast, vΔE296R virus growth showed higher sensitivity to H_2_O_2_ (*p*-value = 0.0036), supporting a role of the viral AP endonuclease in the repair of single-strand breaks with 3′-blocking termini. In eukaryotic systems exposure to *t*-BuO_2_H has been shown to result in a dose-dependent increase of single-strand breaks in DNA (Aherne and O'Brien, 2000; Guidarelli et al., 1997). Exposure to *t*-BuO_2_H results in higher inhibition of vΔE296R replication, as compared to wild type virus (*p*-value = 0.0044). This effect corroborates the role of pE296R in the repair of DNA strand breaks in the viral genome, and may suggest the involvement of the NIR pathway in the drug-resistance. The fact that the ASFV genome encodes an AP endonuclease as well as a DNA repair polymerase and DNA ligase, but not for a DNA glycosylase, is in line with the existence of such a route in the infected cell that might operate, in some cases, as an alternative to a viral BER system. As mentioned above, MMS-induced DNA damage is repaired by BER in prokaryotic and eukaryotic systems (Cunningham et al., 1986; Sobol et al., 1996). Therefore, the finding that exposure to MMS dramatically reduces the titers of vΔE296R more than in the wild type virus (*p*-value = 0.037), indicates that processing of AP sites through the BER pathway is also involved in viral genome maintenance.

Interestingly, we have shown that the production of vΔE296R in swine macrophages strongly decreases in the absence of any treatment (Redrejo-Rodríguez et al., 2006) whereas none of the genotoxic compounds tested decreased the production of vΔE296R in Vero cells to the same extent as in macrophages. This suggests that in the cytoplasm of the infected host macrophage the pE296R protein counteracts various types of DNA damage. Nevertheless, the sensitivity found in Vero cells in the presence of controlled amounts of oxidizing and alkylating agents proves the requirement of DNA repair functions of protein pE296R for viral growth. These results, along with the biochemical properties of the enzyme, support an essential role of the ASFV AP endonuclease in viral DNA repair pathway(s) to eliminate oxidative DNA base and sugar damage and strand breaks *in vivo*, as well as to process AP sites.

## Materials and methods

### Chemicals

Ammonium persulfate, H_2_O_2_, DTT, β-ME, MMS, *t*-BuO_2_H and 1,10-phenanthroline were obtained from Sigma-Aldrich Chimie S.a.r.l. (Lyon, France) and AMS from Molecular Probes Europe (Leiden, The Netherlands).

### Oligonucleotides

Oligonucleotides were purchased either from Eurogentec (Seraing, Belgium) or from Sigma-Genosys (Madrid, Spain). The sequences of the oligonucleotides used for all assays are shown in Table 1. Oligonucleotides were 5′-end labelled with T4 polynucleotide kinase (New England Biolabs) and [γ-^32^P]ATP. The purified 5′-^32^P-labelled oligonucleotides were annealed to complementary nonlabelled oligonucleotides in a buffer containing 60 mM NaCl and 20 mM Tris–HCl (pH 8.0) at 65 °C for 3 min and then slowly cooled to room temperature. The resulting duplex is referred to as X⋅Y, where X is either regular A, C, T and G bases, or the modified residues: THF, DHU, 5ohC, 8oxoG, I, αdA, ɛC and ɛA, whereas Y is an opposite regular base in the complementary strand. For the binding assays, we used the oligonucleotide RT-S with an abasic site bound by a 5′-phosphothioate to the adjacent nucleotide. For 3′-phosphatase, 3′-repair diesterase and 3′ → 5′ exonuclease assays we used nicked duplex oligonucleotide containing a blocking group at 3′ side and a phosphate at 5′ side of the single-strand break. The double stranded substrate used in each experiment is specified in the corresponding figure.

### *E. coli* strains, plasmids and enzymes

*E. coli* AP endonuclease mutant strain BH110 (*nfo::kan*^*R*^ [Δ*(xth-pncA)90 X:Tn10*]) (DE3) and Nfo expression vector (pET11_A_-nfo) were previously described in Ishchenko et al. (2006). The plasmid pRSET_A_-E296R, encoding the histidine-tagged E296R protein, was described in Redrejo-Rodríguez et al. (2006). Control plasmid pRSET_A_ was purchased from Invitrogen.

Human APE1 was purified by the method described in Ishchenko et al. (2006). ASFV AP endonuclease protein (pE296R) was purified as described in Redrejo-Rodríguez et al. (2006) with some modifications. Briefly, histidine-tagged protein from pRSET_A_-E296R plasmid was overproduced in *E. coli* BH110 (DE3) and purified by a single step Ni-NTA chromatography. Cells were lysed with a French Press at 18,000 psi in buffer A (50 mM Tris–HCl (pH 7.5)/500 mM NaCl and 10% glycerol). The homogenate was centrifuged at 40,000 ×*g* for 20 min and the supernatant was clarified by filtration (Ø 0.22 μm) and then applied to a 1 ml HisTrap Chelating column (GE Healthcare) pre-charged with 5 mM NiCl_2_ and equilibrated with buffer A. After washing the column with 10 ml of buffer A, the bound proteins were eluted with gradient of 0 to 500 mM Imidazol in buffer A. Fractions containing pE296R were pooled and desalted on 1 ml Sephadex G-25 column equilibrated with a buffer containing 150 mM NaCl and 20 mM Tris–HCl (pH 7.5). The final protein preparation was stored at − 20 °C in 50% glycerol.

### DNA incision assays

The standard assay mixture for AP endonuclease, NIR, 3′-phosphatase, 3′-phosphodiesterase and 3′ → 5′ exonuclease activities (20 μl final volume) contained 0.5 pmol of 5′-[^32^P]-end labelled oligonucleotide duplex, 20 mM Tris–HCl (pH 8.0), 10 mM NaCl, 0.05% NP40, 5 mM MgCl_2_, 5% glycerol and limiting amounts of proteins, unless otherwise stated. Addition of the reducing agents DTT and β-ME to reaction mixture is indicated. Assays were carried at 37 °C for 15 min and products of the reactions were analyzed by denaturing polyacrylamide gel electrophoresis (PAGE) in 0.5× or 1× TBE buffer. Gels were exposed to a Fuji FLA-3000 PhosphorScreen or subjected to autoradiography and analyzed using Image Gauge V3.12 software.

Kinetic parameter determinations were made using eleven different substrate concentrations in a range from 10 to 300 nM. The *K*_M_ and *k*_cat_ were calculated in al least two independent experiments using the Enzyme Kinetics module from GraphPad Prism 5.0. The values given in Results and Discussion correspond to one representative experiment with the standard error of non-linear fitting.

### DNA binding assays by EMSA.

EMSA was performed as described in Masuda et al. (1998) with slight modifications. Briefly, 40 nM of the pE296R protein was incubated for 20 min on ice in a buffer containing 50 mM Tris–HCl (pH 8), 20 μM 1,10-phenanthroline, 0.2 mg/ml BSA, 0.02% NP40 and varying concentrations of DTT. Then, 1 nM of the 5′-labelled RT-S/CompT oligonucleotide duplex was added. The mixtures (20 μl) were incubated on ice for an additional 10 min and supplemented with 1 μl of GelPilot DNA Loading Dye 5 × (Qiagen) before loading onto a pre-running nondenaturing 7% polyacrylamide gel (39:1) of size 200 × 150 × 1.5 mm. The electrophoresis was run in a buffer containing 6 mM Tris–HCl (pH 8.0), 5 mM sodium acetate, and 0.5 mM EDTA, at 12 mA for 3 h at 4 °C. The gels were fixed for 20 min in 25% 2-propanol/10% methanol, dried and subjected to autoradiography at − 80 °C. The amount of DNA present in each band was quantified using QuantityOne software (Bio-Rad).

### Disulfide bond analysis

The presence of disulfide bonds in the recombinant pE296R protein was analyzed by alkylation assay (Hao et al., 2005). Samples were prepared with 2 μg of purified protein in a buffer containing 20 mM Tris–HCl (pH 8), 10 mM NaCl, 0.05% NP40, 5 mM MgCl_2_ and 5% glycerol with or without 100 mM DTT. Protein was precipitated with trichloroacetic acid (TCA, 10% w/v) for 1 h at 4 °C and then recovered by centrifugation for 10 min at 13 000×g. Pellets were washed once with ice-cold acetone, centrifuged and air-dried for 30 min at room temperature and resuspended in 150 mM Tris–HCl (pH 8), 2% SDS with or without 15 mM AMS. After 1 h incubation at room temperature, SDS-PAGE sample buffer with no reducing agent was added and samples were directly loaded onto a 15% SDS-PAGE. To visualize the protein bands the gels were stained with Coomassie Brilliant Blue R.

### Sensitivity of *E. coli* to DNA damage treatments

Drug sensitivity complementation of *E. coli* BH110 strain by the viral *E296R* gene was examined as described in Cunningham et al. (1986) with minor modifications. Cells were transformed by electroporation with the indicated plasmid and plated in LB-Agar media with 150 μg/ml ampicillin and 25 μg/ml kanamycin followed by overnight incubation at 37 °C. Single colonies were then cultured in LB broth with 0.1 mM IPTG at 37 °C until *A*_600_ reached ∼ 0.6. An aliquot was withdrawn to check protein expression by SDS-PAGE (data not shown). For drug treatments, cells from 1 ml of culture were collected by centrifugation, washed once, and resuspended in sterile phosphate buffered saline (PBS). Serial 1/10 dilutions were also made in PBS. Chemical agents H_2_O_2_, MMS and *t*-BuO_2_H were added to 360 μl of molten soft agar (0.6%) supplemented with 0.05 mM IPTG, at 46 °C, followed immediately by 40 μl of each cell dilution, and the mixture was poured onto the surface of 4 ml of LB-agar (1.5%) in 6-well plates (Falcon). Parallel plates with no drug but with 150 μg/ml ampicillin were plated to control the plasmid lost (data not shown). Colonies were scored after 1–2 days of incubation at 37 °C for H_2_O_2_ and MMS and 28 °C for *t*-BuO_2_H-containing media.

### Viral sensitivity to DNA damage agents

Vero cells were obtained from the American Type Culture Collection and grown in Dulbecco modified Eagle Medium (DMEM) containing 10% fetal calf serum (FCS). Cell infections were made with ASFV BA71V strain and mutant virus lacking the *E296R* gene as previously reported (Redrejo-Rodríguez et al., 2006). Cells were plated at approximately 10^5^ cells per cm^2^ and infected at a multiplicity of infection of 2 pfu/cell in DMEM plus 2% FCS and streptomycin/penicillin G. After 1 h for viral adsorption, the medium was withdrawn and the cells washed with DMEM. Fresh medium containing or not H_2_O_2,_ MMS or *t*-BuO_2_H at varying concentrations was added and maintained until total cytophatic effect (about 48 hpi) was observed. Viral replication is estimated by total viral production calculated by plaque assay titrations (Enjuanes et al., 1976). The BA71V and vΔE248R titers were analyzed by a two-sample paired *t*-test with GraphPad Prism 5.0 in order to determine the statistical significance of the difference between the data.

## Figures and Tables

**Table 1:** Sequence of the oligonucleotides used in the study.

Oligonucleotide name	Sequence^a^
RT-X	5′-TGACTGCATA**X**GCATGTAGACGATGTGCAT-3′
RT-S^b^	5′-TGACTGCATA**S**GCATGTAGACGATGTGCAT-3′
Comp-Y	5′-ATGCACATCGTCTACATGC**Y**TATGCAGTCA-3′
Exo-X	5′-GTGGCGCGGAGACTTAGAGA**X**-3′
5P-19	5′(PO_4_)-ATTTGGCGCGGGGAATTCC-3′
Rex-Y	5′-GGAATTCCCCGCGCCAAAT**Y**TCTCTAAGTCTCCGCGCCAC-3′

a X is either regular A, C, T and G bases, or the modified residues: THF, DHU, 5ohC, 8oxoG, I, αdA, ɛC and ɛA. Y is a corresponding regular base in the complementary strand.

b The S indicates an abasic site bound by a 5′-phosphothioate to the adjacent sugar.
